# Construction and Evaluation of the Bidirectional Referral System in Internet Hospital: Case Study of Children’s Hospital in Western China

**DOI:** 10.2196/69765

**Published:** 2025-07-21

**Authors:** Qinling Li, Yunzhen Deng, Xiangdong Yin, Yingliang Li, Lan Hu, Bin Yang

**Affiliations:** 1 Department of Medical Affairs, Children's Hospital of Chongqing Medical University, National Clinical Research Center for Child Health and Disorders, Ministry of Education Key Laboratory of Child Development and Disorders, Intelligent Application of Big Data in Pediatrics Engineering Research Center of Chongqing Education Commission of China, Chongqing, China; 2 Department of Information, Children's Hospital of Chongqing Medical University, National Clinical Research Center for Child Health and Disorders, Ministry of Education Key Laboratory of Child Development and Disorders, Chongqing Key Laboratory of Pediatric Metabolism and Inflammatory Diseases, Chongqing, China; 3 Department of Pharmacy, Children's Hospital of Chongqing Medical University, National Clinical Research Center for Child Health and Disorders, Ministry of Education Key Laboratory of Child Development and Disorders, Chongqing Key Laboratory of Pediatric Metabolism and Inflammatory Diseases, 136 Zhongshan 2nd Road, Yuzhong District, Chongqing, 400014, China, 86 13618373005

**Keywords:** internet bidirectional referral, internet hospital, children, referral efficiency, traditional bidirectional referrals

## Abstract

**Background:**

The World Health Organization (WHO) emphasizes internet IT as pivotal for optimizing health care system efficiency. Traditional bidirectional referral (TBR) systems, hindered by manual processes and information asymmetry, face challenges in pediatric care. While internet bidirectional referral (IBR) systems demonstrate effectiveness compared to TBR methods, comparative performance analyses remain remarkably scarce.

**Objective:**

This study aims to develop a systematic and standardized bidirectional referral framework for internet hospitals and analyze the differences in core referral indicators of referral time and postreferral medical expenses between TBR (2019‐2021) and IBR (2022‐2024) at the Children’s Hospital of Chongqing Medical University.

**Methods:**

This study is a retrospective cohort study that includes patients aged 0‐18 years with chronic diseases and complex cases in both TBR and IBR periods, while excluding emergency cases. We compared the differences between TBR and IBR across multiple dimensions, including demographic characteristics, downward-to-upward transfer ratio, core indicators (referral times and postreferral medical expenses) and referred diseases, and medical departments.

**Results:**

This study included a total of 457 referral cases, with 106 in the TBR group (79 upward and 27 downward, resulting in a downward-to-upward referral ratio of 34.18%) and 351 in the IBR group (329 upward and 22 downward, resulting in a downward-to-upward referral ratio of 6.69%). Compared with the TBR group, the median referral time in the IBR group was significantly shorter (0.90 d vs 2.51 d; *P*<.001), and the median postreferral medical expenses were lower (¥13,091.16 [US $1822.34] vs ¥8380.59 [US $1166.61]; *P*=.01). We observed that in both groups, the respiratory department consistently ranked as the top specialty for upward referrals, with pneumonia emerging as the most prevalent diagnosis for such transfers.

**Conclusions:**

In pediatric care, the IBR system improved referral efficiency and reduced postreferral medical expenses compared with TBR methods, but there is still a low downward referral rate. While the IBR system shows promise and merits widespread adoption, further validation across diverse medical institutions and broader populations is necessary.

## Introduction

The World Health Organization (WHO), in its Global Strategy on Digital Health (2020‐2025), identifies internet IT as a key driver for addressing inefficiencies within health systems [1]. The continuous advancement of digital health technologies presents new opportunities to optimize the allocation of medical resources and enhance the overall efficiency of health systems [1]. Furthermore, these advancements provide solutions to systemic inefficiencies observed within tiered health care systems. Tiered health care aims to establish a rational and systematic health care network that optimally uses resources and ensures that patient care is appropriately managed across different levels of the health care system [2,3].

Bidirectional referral is fundamental to tiered medical systems, facilitating a reciprocal diagnostic and treatment process between primary and higher hospitals. Traditional bidirectional referral (TBR) is primarily recorded manually, characterized by information asymmetry between hierarchical medical institutions and a lack of real-time coordination [4,5]. These limitations inherently compromise referral efficiency, consequently leading to potential treatment delays for patients.

The rapid development of internet medical technology, driven by policy initiatives such as the “Healthy China 2030” blueprint [6], has provided new opportunities for the transformation and upgrading of health care services, particularly in the application of internet bidirectional referral (IBR). Based on Donabedian’s structure-process-outcome’ model [7], the IBR system, empowered by the integration of internet and ITs, has been structurally improved—for example, through the integration of hospital information systems (HIS)—thereby directly optimizing the referral process by shortening referral times and ultimately improving patient outcomes, including cost reduction.

Existing research on bidirectional referral has predominantly focused on adult populations [8]. However, the pediatric health care landscape is characterized by parental decision-making dynamics rather than the autonomous self-determination seen in adult patients. In addition, the disease spectrum and pharmacological treatment protocols in pediatrics differ significantly from adult health care [9]. There is a notable research gap regarding the applicability of bidirectional referral systems in managing pediatric chronic diseases and complex cases. Moreover, existing studies on TBR and IBR have predominantly focused on theoretical discussions, lacking systematic empirical comparisons based on multidimensional core indicators such as referral time and postreferral health care expenditures (PHCE) [8,10], limiting evidence-based policy formulation.

This study addresses this gap by analyzing real-world data from the Children’s Hospital of Chongqing Medical University, spanning a 6-year period from 2019‐2021 (TBR period) and 2022‐2024 (IBR period). We aim to quantitatively demonstrate the potential of the IBR in reducing referral times and PHCE compared to TBR, while also providing evidence from China to inform global digital health implementation.

## Methods

### Operating Environment and Process of the IBR System

Our internet hospital platform supports a comprehensive bidirectional referral system, enabling all operations including upward and downward referrals, and remote consultations through the “Chongqing Medical University Children’s Hospital” WeChat (Tencent Holdings) public account, WeChat miniprogram, and official website portal. The IBR platform integrates seamlessly with the HIS and collaborative electronic signature mechanism, facilitating real-time patient information transmission, hospital appointment and bed allocation, appointment update push notifications, and consulting physicians’ electronic signatures, with full visualization of referral process progression.

The daily operational management of IBR services is conducted by the Internet Office under the Medical Affairs Department, with collaborative support from the Information Center, Quality Management Department, Medical Insurance Office, and Finance Department. The hospital president serves as the direct project supervisor.

Each collaborating primary hospital designates a dedicated remote liaison officer to ensure smooth referral processes. The primary responsibilities of these liaison officers include communication and coordination with the higher hospital, medical staff, and patients at their respective institutions. Through close collaboration among liaison officers, efficient and accurate communication during the referral process can be guaranteed, enabling timely referral appointments between hierarchical medical institutions.

### Upward Referral Processes Between IBR and TBR

The upward referral process for IBR illustrated in Multimedia Appendix 1 is presented as follows: (1) patients receive initial consultation and preliminary diagnosis and treatment at a primary hospital. When the condition exceeds the hospital’s diagnostic and treatment capabilities, the primary hospital physician submits a remote internet consultation request to the higher hospital, extracts patient-related information from the hospital’s HIS to the IBR platform. (2) Higher and primary hospital physicians conduct remote video consultation at the predetermined time. (3) Following the consultation, the higher hospital physician issues a consultation opinion report. (4) Based on the remote consultation recommendations, the primary hospital discusses potential upward transfer with the patient’s family. If the family decides to proceed with the transfer, they are informed about the referral details and sign the “Bidirectional Referral Informed Consent Form” in the IBR. (5) The physician logs into the IBR system, completes the referral documentation, and submits the upward transfer application to the higher hospital. This referral initiation time is recorded as T1. (6) The higher hospital’s Internet Office reviews the referral application and generates an electronic outpatient or inpatient appointment voucher. (7) For outpatient transfers, patients directly proceed to the higher hospital’s outpatient department on the scheduled consultation day after Internet Office verification. For inpatient transfers, patients are directly admitted to the higher hospital via the green referral channel. This time of successful consultation is recorded as T2.

The upward referral process for TBR illustrated in Multimedia Appendix 2 is presented as follows: (1) patients receive initial consultation and preliminary diagnosis and treatment at a primary hospital. (2) When the patient’s condition exceeds the primary hospital’s diagnostic and treatment capabilities, the hospital physician discusses potential upward transfer with the patient, who subsequently consents to the transfer. (3) The primary hospital contacts the higher hospital’s Medical Insurance Department via telephone to request a patient referral. This referral initiation time is recorded as T1. (4) For outpatient transfers, patients independently proceed to the higher hospital’s outpatient department for registration and consultation. For inpatient transfers, patients independently register at the outpatient department, obtain a hospital admission request, and await bed availability. The time of successful consultation is recorded as T2.

### Downward Referral Processes Between IBR and TBR

The downward referral process for IBR illustrated in Multimedia Appendix 3 is presented as follows: (1) after stabilization following acute treatment at the higher hospital, the patient’s condition becomes stable. (2) The medical team from the higher hospital assesses the patient’s condition. The physician obtains the patient’s consent regarding potential downward transfer. If the patient does not consent to transfer, treatment continues at the higher hospital. Upon the patient’s agreement to transfer, the higher hospital physician logs into the IBR platform, fills out the referral documentation, and submits the downward referral application. This referral initiation time is recorded as T1. (3) The primary hospital physician reviews the referral application in the IBR. (4) The patient obtains the transfer document and proceeds to the Medical Insurance Office to complete settlement and transfer procedures. (5) The patient directly proceeds to the primary hospital for continued rehabilitation treatment via the green channel. The time of successful consultation is recorded as T2. (6) In the event of any condition changes, the higher hospital physician conducts remote rounds to guide the primary hospital in adjusting the treatment plan.

The downward referral process for TBR illustrated in Multimedia Appendix 4 is presented as follows: (1) after completing the acute treatment phase at the higher hospital, the patient’s condition stabilizes. (2) The physician obtains the patient’s consent regarding potential downward transfer. (3) If the patient does not consent to transfer, treatment continues at the higher hospital. (4) Upon the patient’s agreement to transfer, the clinical department notifies the Hospital Admission Management Department. (5) The Admission Management Department contacts the primary hospital. This referral initiation time is recorded as T1. (6) The primary hospital prepares a bed for the patient. (7) The patient is discharged and independently proceeds to the primary hospital for continued treatment. The time of successful consultation is recorded as T2.

### Key Differences in Referral Processes Between TBR and IBR

We conduct a comparative analysis of TBR and IBR across 6 dimensions: remote consultations, methods of information sharing, referral application submission processes, bed arrangement methods, patient informed consent, and postreferral patient management. As for consultations before initiating a referral, TBR lacks this functionality, IBR allows for remote consultations between higher hospitals and primary hospitals regarding the patient’s condition. Regarding information sharing, TBR primarily relies on paper records or telephone communication, IBR enables synchronized sharing of patient condition data through the HIS and the IBR system. As for the referral application process, TBR typically involves communication via telephone, requiring patients to carry paper medical records, but IBR facilitates communication and submission between higher hospitals and primary hospitals through the IBR system, eliminating the need for patients to travel unnecessarily. For bed arrangement, TBR requires patients to make appointments independently, while IBR allows patients to directly access higher hospitals through a green channel for consultations. As for patient informed consent, TBR relies on verbal communication from the physician or the signing of paper documents, while IBR uses electronic signatures. Regarding postreferral management of the patient’s condition, in TBR, higher hospitals no longer participate in the management of the patient’s condition, while in IBR, higher hospitals can conduct follow-up management of the patient’s condition through remote ward rounds.

### Effectiveness Evaluation of IBR vs TBR

#### Study Design

This retrospective study compared TBR (2019‐2021) with IBR (2022‐2024) at the Children’s Hospital of Chongqing Medical University.

#### Inclusion and Exclusion Criteria

Participants aged 0‐18 years who underwent referrals, such as those for chronic disease management or complex cases, at the Children’s Hospital of Chongqing Medical University during TBR (2019‐2021) and IBR (2022‐2024) were selected. Emergency referrals, including acute trauma and critical illnesses transferred via the 120 Emergency system, were excluded. Data collected included patient identification number, sex, diagnosis, referring department, referral initiation time, admission time, discharge time, and direct medical costs within the referral department. Cases with missing data for key variables, including referral initiation time, admission time, or direct medical costs were excluded from the analysis.

#### Definitions of Core Indicators

The core indicators of this study included referral time and PHCE. Referral time is defined as the interval between the confirmation of patient transfer from the referring hospital and the patient’s admission to the receiving hospital’s outpatient clinic or inpatient ward. This is calculated as T2 minus T1 in the referral process. PHCE is defined as the direct medical costs incurred by patients at the receiving medical institution following referral, such as outpatient consultation fees and inpatient treatment expenses. PHCE is expressed in Chinese Yuan (¥).

#### Data Sources and Collection

In the TBR group, data were relied on standard paper-based documentation. In contrast, the IBR system enabled the sharing of patients’ previous medical information. All collected data underwent rigorous data cleaning and organization to ensure the accuracy and validity of the analysis results.

### Statistical Analyses

Analysis was performed using Python 3.8 with scientific computing libraries. Descriptive statistics were calculated for all variables. After testing for normality (Shapiro-Wilk test) and homogeneity of variances (Levene test), nonparametric Mann-Whitney *U* tests were used to compare age distribution, referral time, and PHCE between groups. Effect sizes were calculated using Cohen *d*. This study employed multivariate linear regression analysis with referral time and PHCE as dependent variables, referral group (TBR vs IBR) as the primary independent variable, age (in months), and sex as covariates to control for potential confounding factors inherent in nonrandomized trials. Statistical significance was set at *P*<.05 (2-tailed).

### Ethical Considerations

Ethical approval (approval file number: 2025.167) was obtained from the Ethics Committee of Children’s Hospital of Chongqing Medical University. This study involved secondary analysis using pre-existing records. Consequently, no additional informed consent was required from participants. And it did not involve the recruitment of living subjects for new interventions or data collection activities. Therefore, no compensation was provided to participants. All data collected during the study were deidentified before analysis to ensure the privacy and confidentiality of participant information. No personally identifiable information was retained or disclosed at any stage of the research.

## Results

### Study Population Description

**Figure 1.**
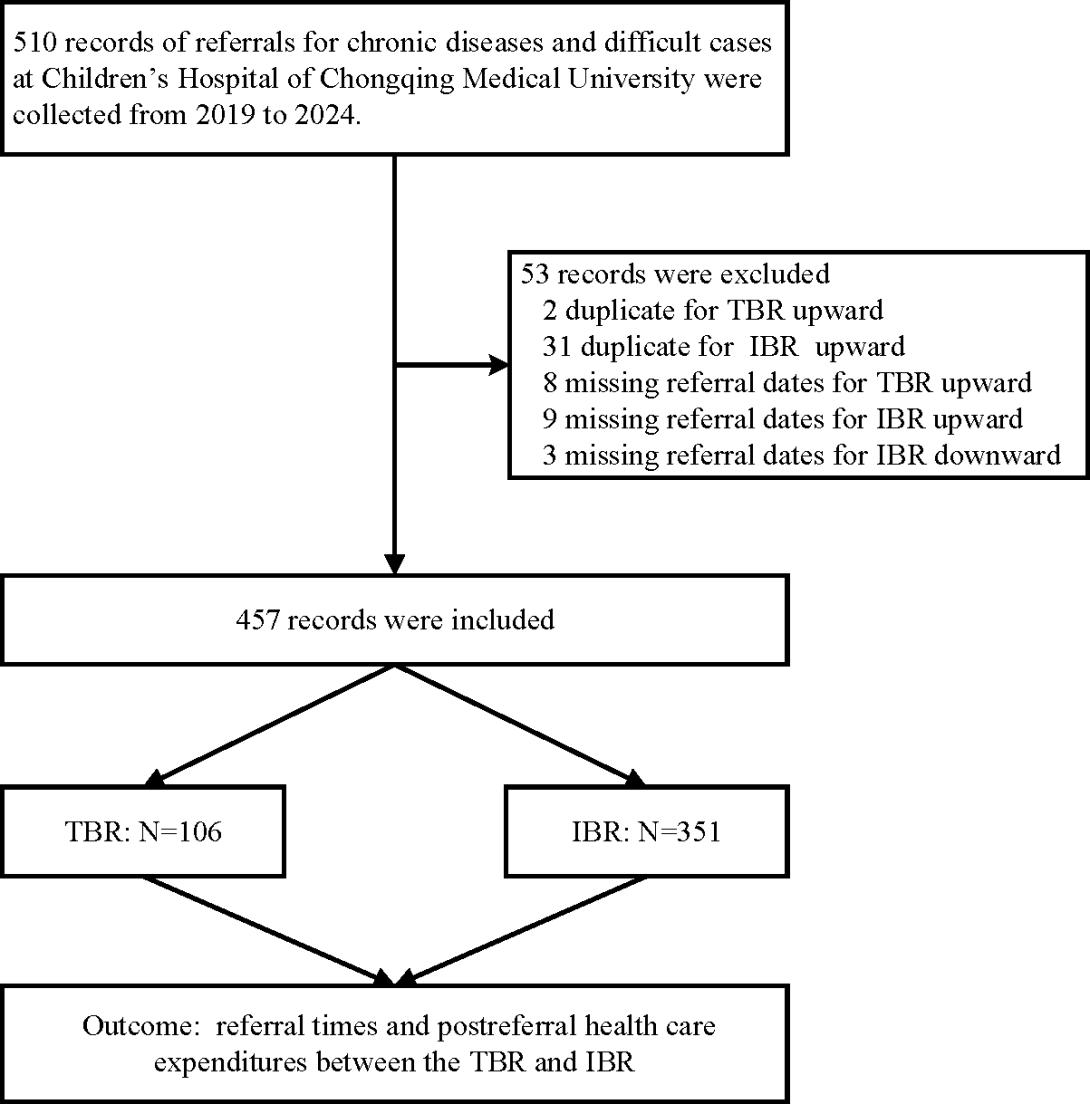
Study flowchart. IBR: internet bidirectional referral; TBR: traditional bidirectional referral.

The distribution of patients in terms of number, sex, and age (in months) for the TBR group and IBR group was shown in Table 1. For sex distribution, the TBR group had 64 (60.38%) males and 42 (39.62%) females, whereas the IBR group had 191 (54.42%) males and 160 (45.58%) females. The *P* value for sex distribution between the 2 groups was .28. The median age for the TBR group was 20.50 months, with an IQR of 7.00-56.75 months. In contrast, the median age for the IBR group was 45.00 months with an IQR of 10.00-84.00 months. The *P* value for age was .002. The study flowchart is shown in Figure 1.

**Table 1.** Demographic characteristics of patients in traditional bidirectional referral versus internet bidirectional referral.

Variables	TBR^a^ group	IBR^b^ group	*P* value
Sex, n (%)			.28
Male	64 (60.38)	191 (54.42)	
Female	42 (39.62)	160 (45.58)	
Age (months）	20.50 (7.00‐56.75)	45.00 (10.00‐84.00)	.002

a TBR: traditional bidirectional referrals.

b IBR: internet bidirectional referrals.

### Overview of Referrals

**Figure 2.**
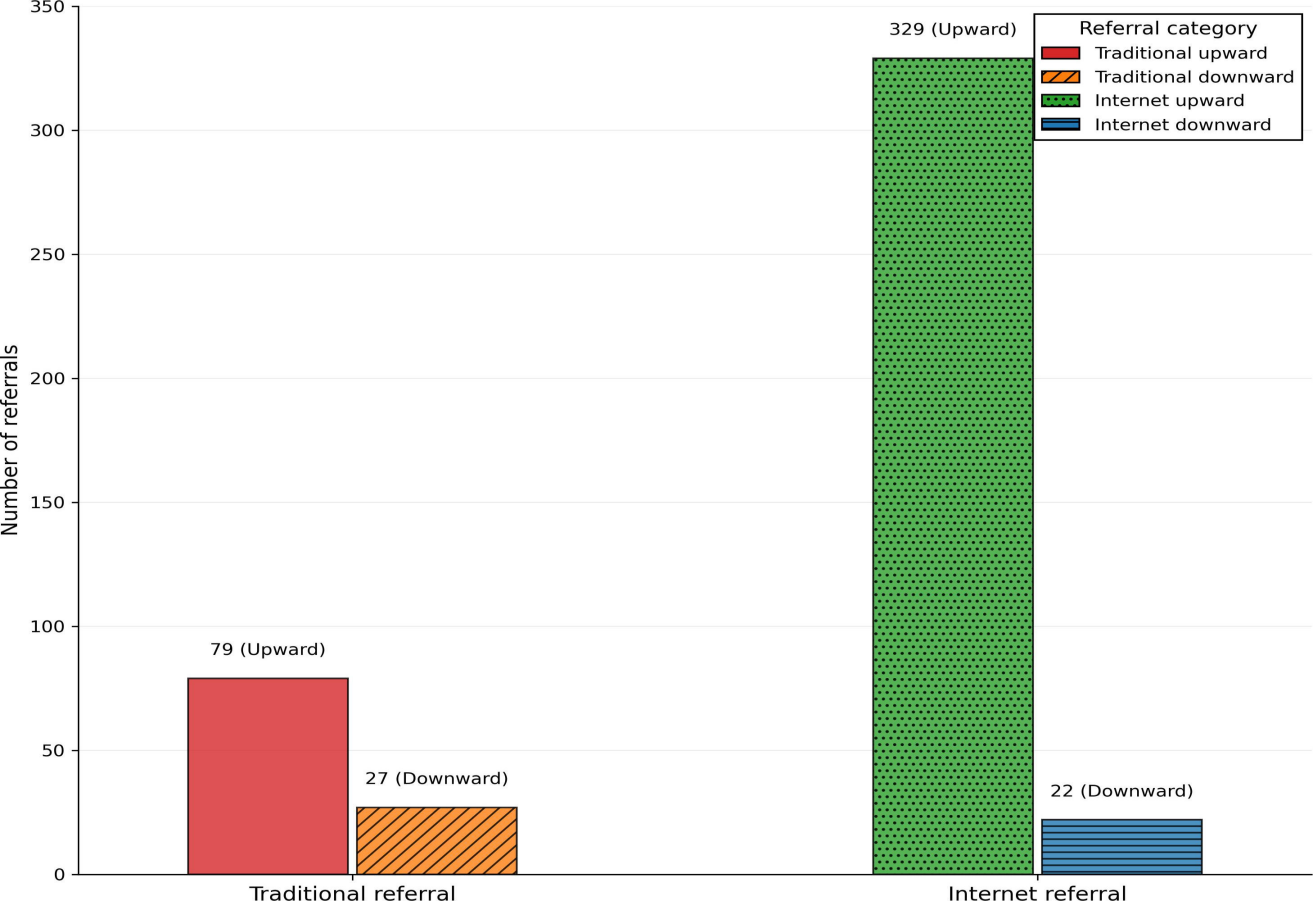
Comparison of upward and downward referral frequencies in traditional bidirectional referral vs internet bidirectional referral.

A total of 457 patient referrals were included in this study, with 106 (23.2%) in the TBR group and 351 (76.8%) in the IBR group as shown in Figure 2. In the TBR group, there were 79 upward transfers and 27 downward transfers, resulting in a downward-to-upward transfer ratio of 34.18%. In contrast, within the IBR group, there were 329 upward transfers and 22 downward transfers, yielding a downward-to-upward transfer ratio of 6.69%.

### Comparison of Referral Time and PHCE Between TBR and IBR

The Shapiro-Wilk test indicated non-normal distribution of both referral duration and PHCE in both groups. Levene test showed homogeneous variances between groups for both variables (referral time: W=2.40, *P*=.12; PHCE: W=3.42, *P*=.07). Due to non-normal distributions, Mann-Whitney *U* tests were used for between-group comparisons. For referral time, the Mann-Whitney U test revealed a statistically significant difference between TBR and IBR (U=18,330.5; *P*<.001). The median referral duration was significantly shorter in the IBR group (0.90 days, IQR 0.75-1.66) compared to the TBR group (2.51, IQR 0.66-6.51 days; *P*<.001; Table 2). For PHCE, the Mann-Whitney *U* test also revealed a statistically significant difference between groups (U=21,533.5; *P*=.01). The median medical cost was significantly lower in the IBR group (¥8380.59 [US $1166.61], IQR ¥5913.27 [US $823.15] to ¥13,641.86 [US $1899.00]) compared with the TBR group (¥13,091.16 [US $1822.34], IQR ¥7040.68 [US $980.09] to ¥19,548.18 [US $2721.18]; *P*=.01). The effect size for cost difference (Cohen *d*=−0.21) suggests an average cost saving of ¥4710.57 (US $655.73) per referral with the internet approach. Multimedia Appendix 5 presented the results of the multivariable regression analysis for referral time and PHCE.

**Table 2.** Comparison of core indicators between traditional bidirectional referrals and internet bidirectional referrals.

Variables	TBR^a^ (n=79)	IBR^b^ (n=329)	*P* value
Referral time (day)	2.51 (0.66‐6.51)	0.90 (0.75‐1.66)	<.001
PHCE^c^ (¥)	13091.16 (7040.68‐19548.18)	8380.59 (5913.27‐13641.86)	.01

a TBR: traditional bidirectional referrals.

b IBR: internet bidirectional referrals.

c PHCE: postreferral health care expenditures.

### Distribution of the Top 5 Departments in Upward Referrals

The distribution features of the top 5 departments under the 2 referral patterns are as follows. In the IBR group (n=329), the Respiratory Department ranked first with 126 (38.30%) referrals. The Neurology Department was second with 49 (14.89%) referrals, followed by the Neonatal Department with 23 (6.99%) referrals in third place, the Gastroenterology Department with 21 (6.38%) referrals in fourth place, and General Practice with 17 (5.17%) referrals in fifth place. In the TBR group (n=79), the Respiratory Department also ranked first with 22 (27.85%) referrals. The Infectious Disease and Neurology Departments tied for second place with 9 (11.39%) referrals each. The Neonatal Department had 5 (6.33%) referrals and ranked fourth, and the Gastroenterology Department had 4 (5.06%) referrals and ranked fifth.

### Distribution of the Top 5 Diagnoses in Upward Referrals

The distribution features of the top 5 diagnoses under the 2 referral patterns are as follows. In the IBR upward referral group, there were 2 missing cases of diagnostic information, while in the TBR upward referral group, there were 6 missing cases. In the IBR group, pneumonia ranked first with 106 (32.42%) referrals. Bronchitis ranked second with 13 (3.98%) referrals, followed by premature infants in third place with 10 (3.06%) referrals. Epilepsy ranked fourth with 7 (2.13%) referrals, and wheezing under investigation came in fifth with 6 (1.82%) referrals. In the TBR group, pneumonia also ranked first with 18 (24.66%) referrals, and developmental delay ranked second with 4 (5.48%) referrals. Cerebral palsy, traumatic subarachnoid hemorrhage, and bronchial asthma each had 2 (2.73%) referrals and ranked third jointly.

## Discussion

### Principal Findings

This study, through a 6-year retrospective cohort analysis based on the example of the Children’s Hospital of Chongqing Medical University, is the first to compare the effectiveness of TBR with the IBR model. The results indicate that, compared with TBR, IBR statistically significantly improved in referral efficiency (1.61 d reduction; *P*<.001) and cost reduction (¥4710.59 [US $655.73]; *P*=.01), alongside a surge in upward referrals (79-329 cases). However, the downward-to-upward referral ratio decreased from 34.18% to 6.69%. And the effect sizes (Cohen *d*=–0.21 for costs) represent small-to-moderate impacts, indicating that while the improvements are measurable, the practical implications of these gains must be contextualized and their clinical or operational significance may depend on institutional priorities.

The efficiency gains of IBR may be attributed to structural optimizations such as streamlined referral workflows, real-time data sharing, and automated appointment scheduling, which align with previous theoretical assertions about digital referral systems [8,10,11]. In addition, the reduction in PHCE may be achieved through the sharing of patient information between primary and higher hospitals within the IBR system, thereby preventing unnecessary re-examinations for patients [12]. Nevertheless, these benefits should be interpreted cautiously, as they primarily reflect operational improvements within a specialized pediatric setting.

Despite these improvements, this study focused primarily on core efficiency metrics—referral time and PHCE—but patient-centered outcomes, such as patient satisfaction and clinical success rates, are equally essential for a comprehensive evaluation of the value of IBR. While these dimensions were not directly measured in our study, previous research suggests that improvements in efficiency may indirectly enhance patient experience [13]. For example, reduced waiting times and alleviation of financial burden are closely associated with higher patient satisfaction scores. A streamlined referral process can also improve treatment adherence by reducing the stress experienced by families during hospital-to-hospital transitions. Future implementations of IBR should integrate patient-centered outcomes [14], including satisfaction or treatment success.

Despite its focus on pediatric chronic care, the structural improvements of the IBR system—such as promoting the shared use of medical resources, automated appointment scheduling, and optimizing the referral process—are technology-driven interventions that transcend age-specific contexts. For example, studies in adult populations have demonstrated similar efficiency gains through comparable digital referral platforms [8], suggesting that the core mechanisms of IBR may be applicable to broader referral needs.

The persistently low downward referral ratio (6.69% in IBR) has been a persistent challenge faced by health care institutions. For example, Zhao et al [15], analyzed the status of the bidirectional system between Sichuan Provincial People’s Hospital and 21 community health service centers in Chengdu from April 2010 to March 2011, finding that the ratio of downward to upward referrals was only 4.94%.

The notably low downward-to-upward referral ratio in the IBR system may be attributed to several factors. First, patients may exhibit a lack of trust in health care providers at primary care institutions, which could contribute to a tendency to seek higher medical care [16-18]. Second, primary care facilities often face limitations in medical equipment and expertise, outdated electronic medical record systems, inadequate integration with the internet, and financial constraints, resulting in insufficient service capacity [19,20]. Third, with regard to medical insurance policies, the reimbursement rates exhibit minimal disparities across different levels of health care institutions, thereby impeding the effective stratification of patient referrals [21]. Due to the absence of unified referral standards, higher medical institutions, driven by financial interests, are also reluctant to facilitate downward referrals for patients [11,22]. In addition, the implementation of the IBR system may have primarily addressed the convenience of upward referrals [3], while falling short of comprehensively resolving the barriers to downward referrals from higher to primary hospitals. Moreover, as a core component of hierarchical medical services, this referral system was introduced during China’s health care reform transition, with the referral model still in an exploratory phase and far from full maturation [23]. Our study period (2019‐2024) coincided with the COVID-19 pandemic, which profoundly impacted global health care service systems and patient health care–seeking behaviors [24,25]. During the pandemic, heightened patient concerns about infection risks further intensified their preference for higher medical institutions. These factors collectively contribute to the observed discrepancies in referral patterns between the 2 systems.

Compared with developed countries, such as the United Kingdom, which has a health care system centered around general practitioners (GPs), patients are required to first consult a GP. The GP then determines whether a referral to a specialist or hospital is necessary based on the patient’s condition [26,27]. In contrast to the mandatory referral model in the United Kingdom, the health care–seeking behavior in China is characterized by greater autonomy, allowing patients to directly choose different levels of medical institutions [28]. Under the internet referral model, the ultimate decision-making power regarding referrals still rests with the patients. This autonomy in medical decision-making may contribute to an insufficient referral rate to lower-tier facilities. Japan’s health care institutions, similar to those in China, are categorized into 3 levels. However, outpatient services at tertiary hospitals have been eliminated, emphasizing the role of primary care facilities. Japan has established strict referral rate standards, such as 60% for upward referrals and 80% for downward referrals, or 40% for upward referrals and 60% for downward referrals. These standards serve as assessment criteria, and hospitals that do not meet these benchmarks are ineligible for financial subsidies, thereby promoting the operation of bidirectional referrals among health care institutions [29,30]. In China, the referral rate of patients currently lacks explicit mandatory regulations. The performance evaluation of tertiary hospitals has only gradually increased its requirements regarding the number of patient referrals [31]. The approach adopted by Japan, which directly constrains hospital referral behaviors through fiscal subsidies, may offer valuable insights for China’s health care system reform.

To address the issue of low downward referral rates in bidirectional referrals, several strategies can be implemented. First, increasing investment in primary health care institutions is essential to enhance their service capacity and attractiveness [32]. Second, strengthening trust-building initiatives with primary care facilities and health education initiatives will help increase public acceptance of tiered medical care and guide residents toward appropriate health care–seeking behaviors [33,34]. Third, establishing standardized downward referral criteria and incorporating downward referral rates as a key performance indicator for clinical departments will create institutional motivation for implementing effective referral strategies [35,36]. At the government level [37], a public data platform can be established, led by the government, to facilitate the sharing and uploading of electronic medical record data and imaging data from health care institutions at all levels [38]. This initiative aims to reduce information barriers and enhance the efficiency of information sharing. In addition, further increasing the reimbursement ratio for primary institutions compared to higher institutions can incentivize more patients to transfer to these primary care facilities [39,40].

Although this study excluded emergency cases, the IBR system continues to play a critical role in emergency situations and pandemics. Through its information-sharing capabilities, remote consultation features, and “green channel” pathways, the IBR system can reduce the time to treatment for critically ill patients and improve the success rate of interventions [41]. During the COVID-19 pandemic, the remote consultation functionality of the IBR system helped minimize patient gatherings at physical health care facilities, thereby reducing the risk of cross-infection [24].

### Limitations

This study has several limitations. First, it was conducted in a single-center, tertiary comprehensive children’s hospital located in Western China, and the exclusive focus on pediatric patients with chronic and complex cases may restrict the generalizability of findings to adult populations or emergency care settings. The pediatric health care landscape is characterized by parental decision-making dynamics, in contrast to adult patients who possess independent self-determination rights. Moreover, the disease spectrum and pharmacological treatment protocols in pediatric medicine differ significantly from adult health care [9]. Given that the data were collected from a specific geographic and demographic group, these may potentially limit the direct generalizability of our findings to other health care institutions and adult populations.

### Future Directions

Future research and implementation should prioritize (1) integrating patient-centered outcomes like satisfaction or treatment success; (2) proposing specific strategies for increasing downward referrals, such as increasing investment in primary health care institutions, trust-building initiatives with primary care facilities, patient education programs, revising medical insurance reimbursement rates to incentivize appropriate-level care seeking, and establishing standardized downward referral criteria; and (3) highlighting the importance of international validation studies to test the system in diverse health care environments and adult populations.

### Conclusions

The IBR system demonstrated significant improvements in reducing referral time, lowering medical costs for patients. However, the system’s effectiveness is constrained by low downward referral rates. While the system shows promise and merits widespread adoption, further validation across diverse medical institutions and broader populations is necessary. This study provides empirical evidence for the deepening of hierarchical medical treatment policies and contributes a distinctively Chinese approach to the global digital health implementation.

## Supplementary material

10.2196/69765 Multimedia Appendix 1 The upward process of internet bidirectional referrals.

10.2196/69765 Multimedia Appendix 2 The upward process of traditional bidirectional referrals.

10.2196/69765 Multimedia Appendix 3 The downward process of internet bidirectional referrals.

10.2196/69765 Multimedia Appendix 4 The downward process of traditional bidirectional referrals.

10.2196/69765 Multimedia Appendix 5 Multivariable regression analysis of referral time and postreferral health care expenditures.

## Abbreviations

GP: general practitioner
HIS: hospital information systems
IBR: internet bidirectional referral
PHCE: postreferral health care expenditures
TBR: traditional bidirectional referral
WHO: World Health Organization
