# Supplementary figures and images for: Construction and Evaluation of the Bidirectional Referral System in Internet Hospital: Case Study of Children’s Hospital in Western China
Source: J Med Internet Res. 2025 Jul 21;27:e69765. doi: 10.2196/69765 (PMC12303232; doi:10.2196/69765)

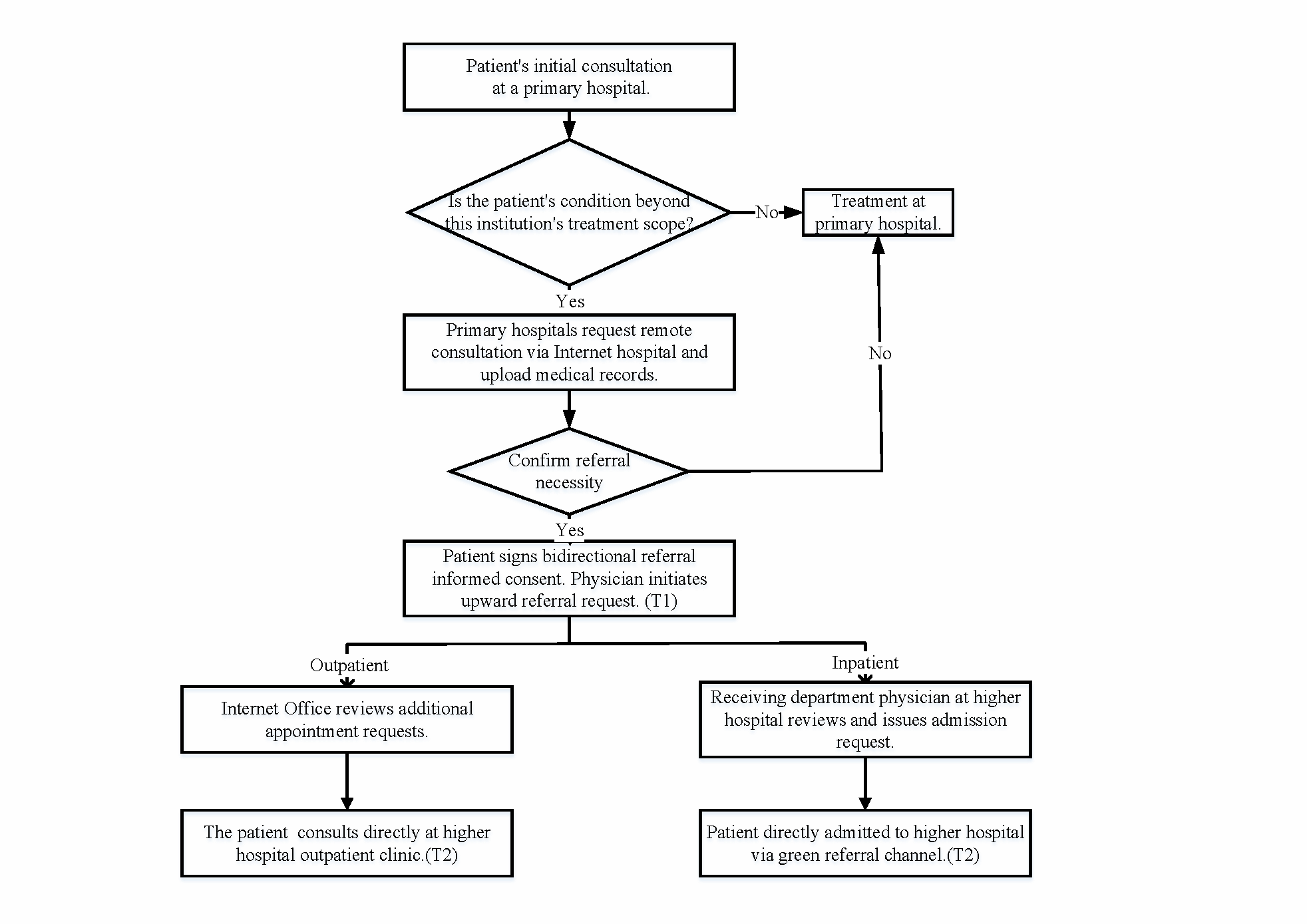

Supplement: Multimedia Appendix 1 [file jmir-v27-e69765-s001.png]

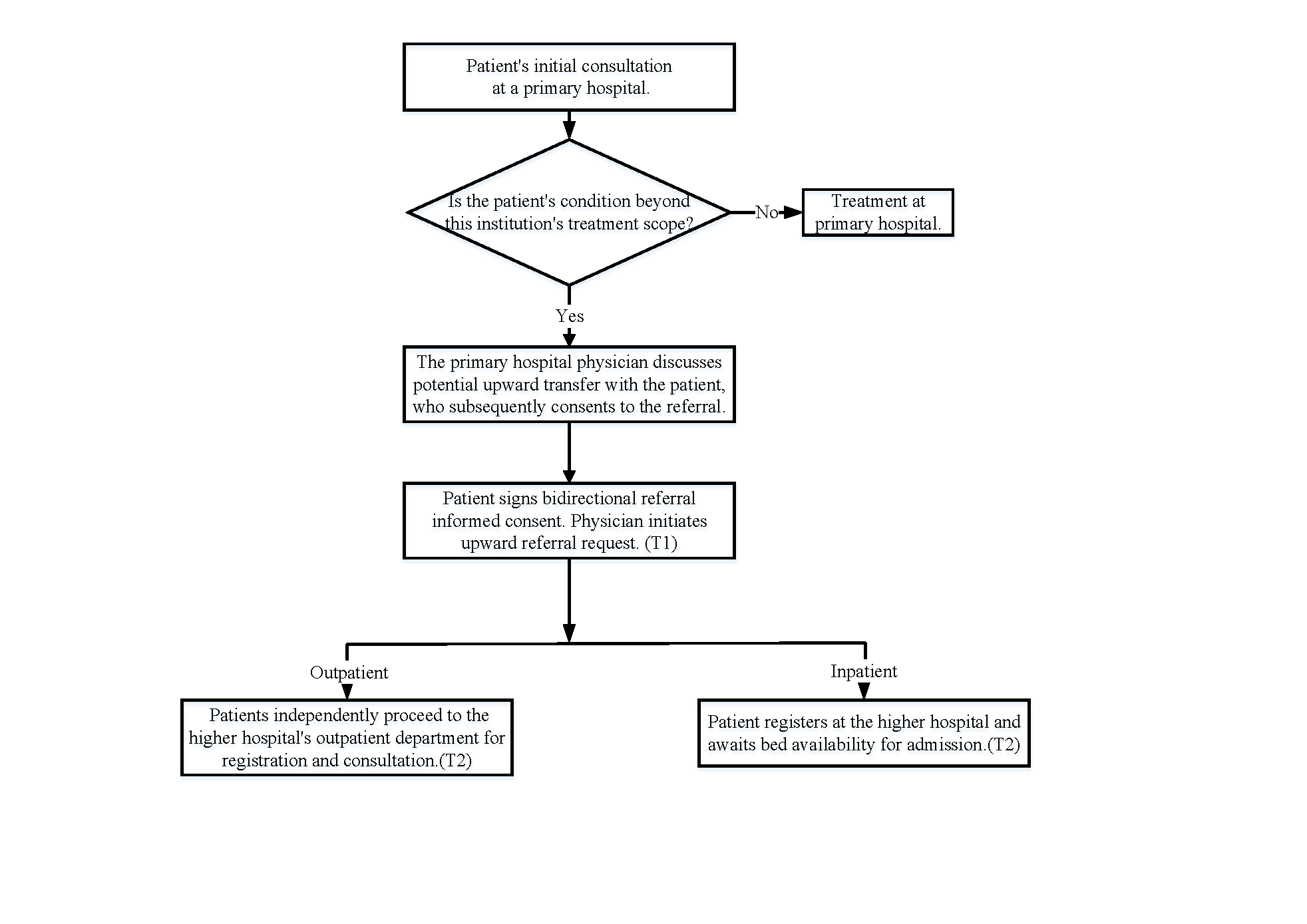

Supplement: Multimedia Appendix 2 [file jmir-v27-e69765-s002.png]

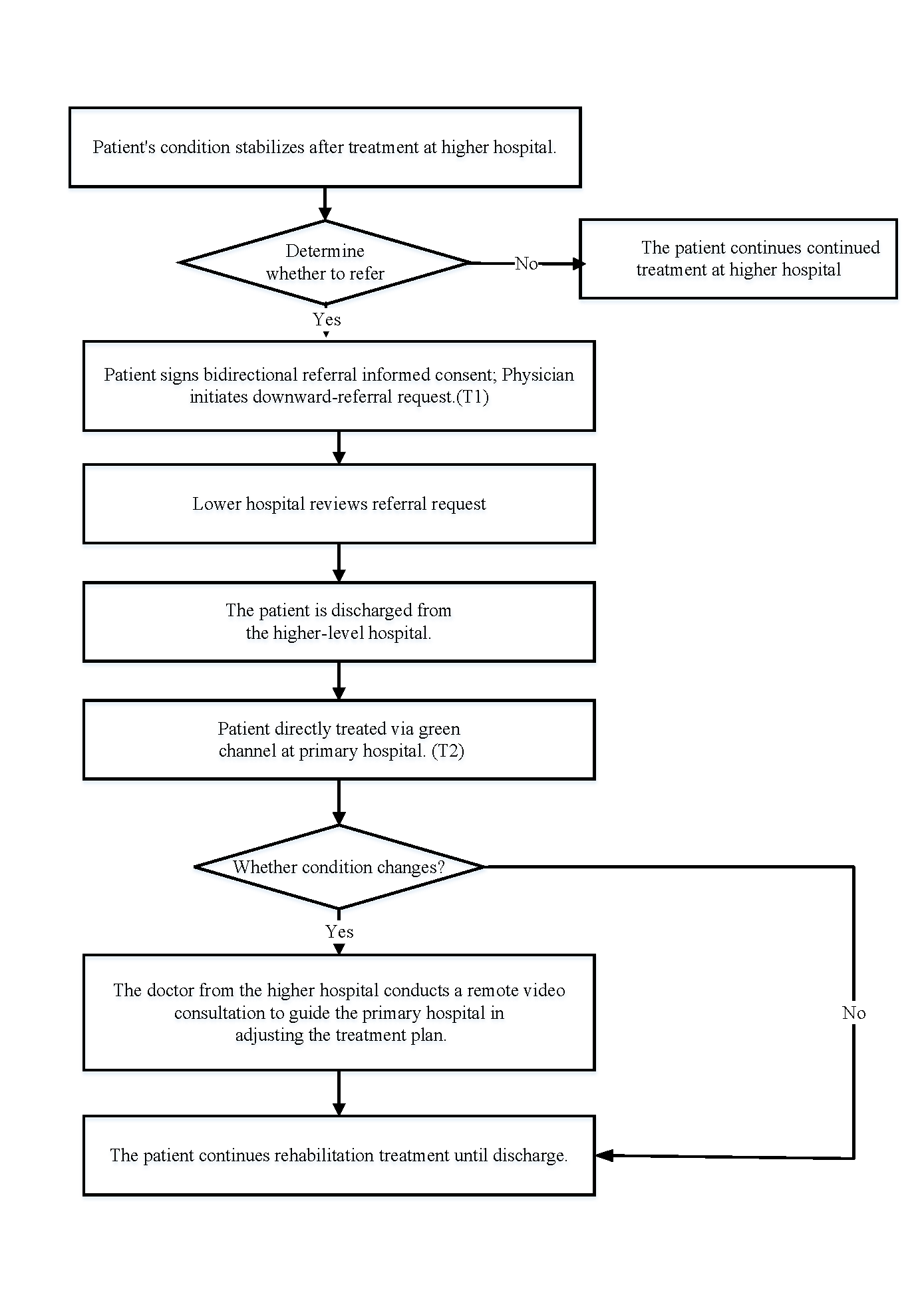

Supplement: Multimedia Appendix 3 [file jmir-v27-e69765-s003.png]

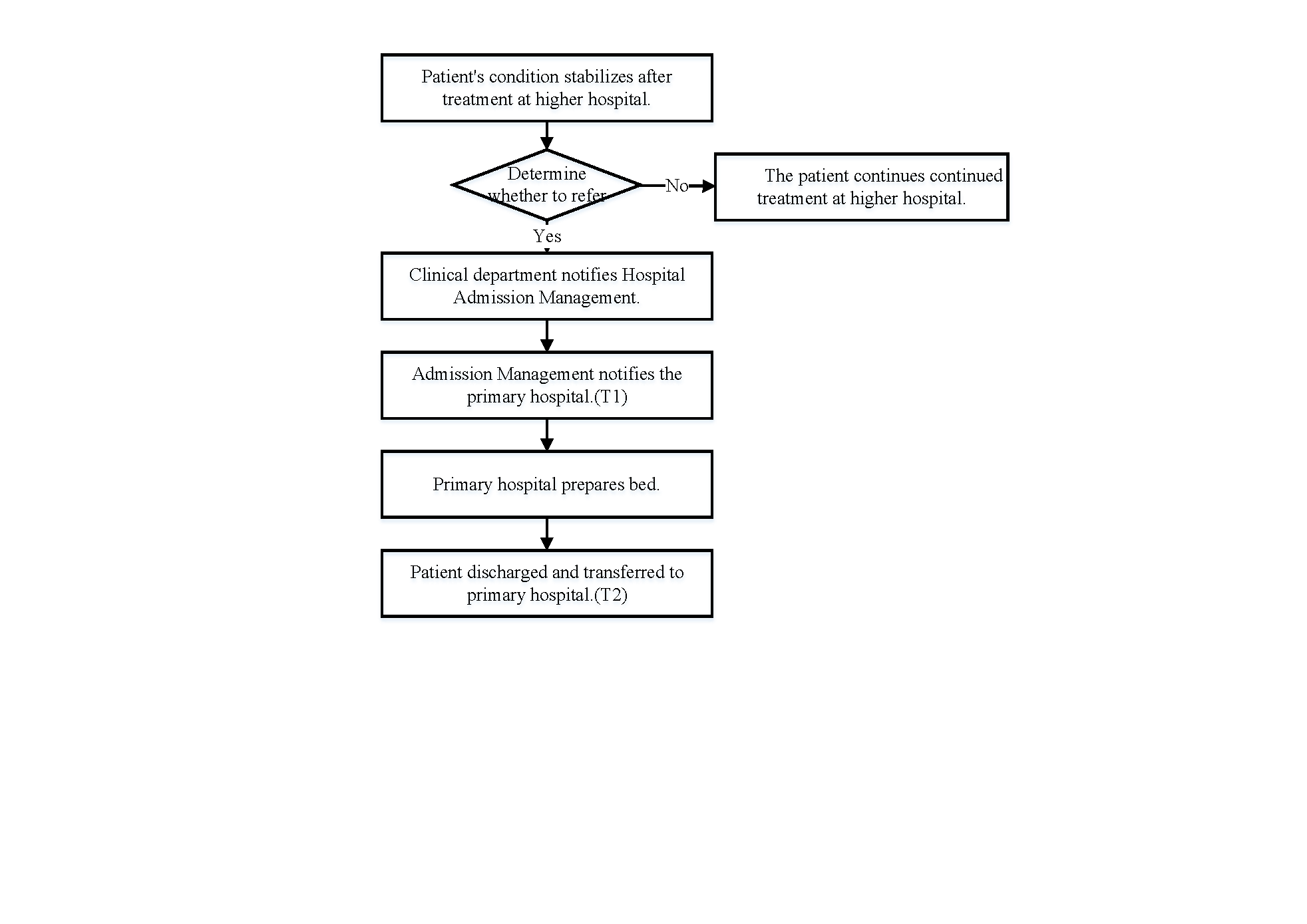

Supplement: Multimedia Appendix 4 [file jmir-v27-e69765-s004.png]
