# Supplementary material for: Construction and Evaluation of the Bidirectional Referral System in Internet Hospital: Case Study of Children’s Hospital in Western China
Source: J Med Internet Res. 2025 Jul 21;27:e69765. doi: 10.2196/69765 (PMC12303232; doi:10.2196/69765)
Supplement: Multimedia Appendix 5 [file jmir-v27-e69765-s005.docx]

**Multimedia Appendix 5.** Multivariable regression analysis of referral time and PHCE.

| Variables | **Referral Time (Days)** | | PHCE ^a^ **(¥)** | | |
| --- | --- | --- | --- | --- | --- |
|  | β (95%Cl) | *P* value | β (95%Cl) | *P* value | |
| TBR^b^ | \| Reference \| \| --- \| | | \| Reference \| \| --- \| | | |
| IBR^c^ | -2.11  (-2.59, -1.62) | <.0001 | -7098.65  (-13950.31, -246.99) | | .043 |

^a^PHCE: postreferral health care expenditures.

^b^TBR: Traditional bidirectional referrals.

^c^IBR: Internet bidirectional referrals.

The multivariable linear regression analysis, adjusted for age and gender, demonstrated significant associations between IBR system and improved referral outcomes compared to TBR. Implementation of the IBR system was associated with a 2.11-day reduction in referral time (β = -2.11, 95% CI: -2.59 to -1.62, *P* < .0001). Similarly, the IBR group showed a ¥7,098.65 decrease in post-referral healthcare expenditures (β = -7,098.65, 95% CI: -13,950.31 to -246.99, *P* = .043).
